# Longitudinally aligned inner-patterned silk fibroin conduits for peripheral nerve regeneration

**DOI:** 10.1007/s44164-023-00050-3

**Published:** 2023-04-18

**Authors:** Ane Escobar, Mariana R. Carvalho, Tiago H. Silva, Rui L. Reis, J. Miguel Oliveira

**Affiliations:** 1 3B’s Research Group, I3Bs—Research Institute on Biomaterials, Biodegradables and Biomimetics, University of Minho, Headquarters of the European Institute of Excellence on Tissue Engineering and Regenerative Medicine, AvePark, Parque de Ciência e Tecnologia, Zona Industrial da Gandra, 4805-017 Barco, Guimarães, Portugal; 2 ICVS/3B’s—PT Government Associate Laboratory, 4710-057 Braga, Braga Portugal; 3 Centro de Física de Materiales (CSIC-UPV/EHU), Paseo Manuel de Lardizabal 5, 20018 Donostia-San Sebastián, Spain

**Keywords:** Silk fibroin conduits, Peripheral nerve regeneration, NGCs

## Abstract

Peripheral nerve injuries represent a major clinical challenge, if nerve ends retract, there is no spontaneous regeneration, and grafts are required to proximate the nerve ends and give continuity to the nerve. The nerve guidance conduits (NGCs) presented in this work are silk fibroin (SF)-based, which is biocompatible and very versatile. The formation of conduits is obtained by forming a covalently cross-linked hydrogel in two concentric moulds, and the inner longitudinally aligned pattern of the SF NGCs is obtained through the use of a patterned inner mould. SF NGCs with two wall thicknesses of ~ 200 to ~ 400 μm are synthesized. Their physicochemical and mechanical characteristics have shown improved properties when the wall thickness is thicker such as resistance to kinking, which is of special importance as conduits might also be used to substitute nerves in flexible body parts. The Young modulus is higher for conduits with inner pattern, and none of the conduits has shown any salt deposition in presence of simulated body fluid, meaning they do not calcify; thus, the regeneration does not get impaired when conduits have contact with body fluids. In vitro studies demonstrated the biocompatibility of the SF NGCs; proliferation is enhanced when iSCs are cultured on top of conduits with longitudinally aligned pattern. BJ fibroblasts cannot infiltrate through the SF wall, avoiding scar tissue formation on the lumen of the graft when used in vivo. These conduits have been demonstrated to be very versatile and fulfil with the requirements for their use in PNR.

**Supplementary Information:**

The online version contains supplementary material available at 10.1007/s44164-023-00050-3.

## Introduction

The nervous system has a limited self-regenerating capacity due to its complex physiology, where the main functional units are neurons [1]. Peripheral nerve repair (PNR) happens due to the numerous growth factors (GFs) that Schwann cells (SCs) secrete and upregulate, such as neurotrophin-3 or neurotrophin-4, the nerve growth factor, glial cell line-derived neurotrophic factor, ciliary neurotrophic factor, and brain-derived neurotrophic factor [2, 3]. Peripheral nerve injuries (PNIs) can happen as a consequence to a trauma and often is related to limb injuries. Injuries of peripheral nervous system (PNS), known as peripheral neuropathies, are a major source of disabilities and occur with a high frequency. PNIs implicate sensory and motor function; sensation can suffer distortion, and a loss in muscle mobility can occur [1, 4].

The time-lapse between the injury and the regeneration of the nerve defines the outcomes of functional recovery, avoiding muscle atrophy [5]. When nerve ends retract, nerves lose their self-regenerating capacity, and surgery is performed to suture nerve ends. However, for nerve injuries longer than 20 mm, this is not possible; due to the gap between nerve ends, they cannot be approximated, and in these cases, grafts are used. Using autografts is one of the more successful methods in terms of PNI recovery, but disadvantages associated with autograft harvesting include secondary surgery requirements, morbidity at the site of injury, and loss of sensory function at the harvesting site, among others. To overcome this, tissue engineering and regenerative medicine (TERM) strategies based on the use of artificial constructs, called nerve guidance conduits (NGCs), are being developed [6, 7]. Such type of conduits aims to bridge the injured nerve ends, protects the regeneration from scar tissue formation and guides the regeneration from the proximal to the distal nerve stump [8]. To promote the growth of the axonal tracks, therapeutic stimulants can be added to the conduits, such as GFs or stem cell–based therapies, with Schwann cells often used as the cell of choice [9, 10]. The porosity, biocompatibility, stiffness/flexibility and suturability of the NGCs are key properties to achieve success in PNR [6, 7].

Several NGCs are commercially available; these include NeuraGen, NeuroFlex, NeuroMatrix, NeuroWrap, NeuroMend, Neurotube and Neurolac, constructed with collagen type I, apart from Neurotube and Neurolac, which are made of polyglycolic acid and poly(l-lactide-co-caprolactone), respectively. These NGCs have shown neuroma formation at the implementation site, and this scenario is even worst while the diameter of the NGC increases [7, 11, 12]. Natural materials have gained the attention of the field because of their biodegradability, biocompatibility and non-toxicity; these include the collagen, gelatin, hyaluronic acid (HA), laminin, fibrin or silk fibroin (SF) [13–22]. Natural-based polysaccharides such as the chitosan (Cht) or alginate have also been studied with good outcomes [23–27]. The natural material-made NGCs can have weak mechanical properties that they have, but the physicochemical properties can be enhanced with the adequate modification of the materials. Crosslinking is the most widely used modification; it links the polymer chains in between them, and the tensile strength or the crush resistance of NGC can be improved [28–31]. SF obtained from *Bombyx mori* (silkworm) has been reviewed as a biomaterial historically recognized for its strength [32]. Silkworms are largely cultivated worldwide, making it a cost-effective material, which makes it suitable for technology transfer [33]. In a previous work of our group, it has been demonstrated the reproducibility of the synthesis of SF NGCs taking advantage of the tyrosine group present in the silk to obtain enzymatically cross-linked hydrogels [34]. The enzyme horseradish peroxidase (HRP) is used, and as substrate hydrogen peroxidase (H_2_O_2_). The HRP/H_2_O_2_ cross-linking method allows the transition from silk solution to a hydrogel, amorphous and transparent and highly stable [35].

In this study, we successfully demonstrate patterned SF-based NGCs synthesis and their successful outcomes for PNI repair. Some previous works support our results, demonstrating that longitudinal patterns, for example in the form of fibres, can promote neurite outgrowth [36–38]. Mechanical studies demonstrated the optimal Young’s modulus of the developed conduits, as well as their kinking and suturability resistance. SF-based NGCs did not show apatite-forming capacity, studied by immersing the conduits are simulated body fluid. In vitro assays with Schwann cells and BJ fibroblasts have proven the biocompatibility of the conduits and the enhanced proliferation of cells when the inner wall has longitudinally aligned patterns. Also, BJ fibroblasts could not infiltrate through the SF walls of the conduit, avoiding their entrance to the lumen without scar tissue formation.

## Results and discussion

### Enzymatically cross-linked SF conduits

The use of tubular structures for regenerative purposes in the TERM field is based on the implantation of engineered conduits. They should mimic the features of the implantation site, which can be soft or hard tissues [39]. We should be able to control the manufacturing parameters in order to obtain reproducible tubular structures. The strategy we are presenting brings us to obtain optically transparent and biocompatible hydrogels due to the presence of 5 mol% tyrosine groups present in the SF, which are covalently cross-linked through a reaction peroxidase/hydrogen peroxidase. The covalent cross-link mediated by the HRP allows us to obtain a more stable and stronger 3D network, if results are compared to conventional methods [35]. The final conduits will have a superior elasticity and mechanical properties, rather than for amorphous conformations. For conduit production, the SF is diluted to 16 wt% and mixed with HRP and H_2_O_2_. This solution is introduced in between two concentric cylinder moulds, some containing inner longitudinally aligned grooves. It is incubated for 30 min at 37 °C to produce the transition to gel state. The system is introduced in liquid N_2_ to obtain a temporary β-sheet state, giving rise to a permanent semicrystalline state which facilitates the removal of the outer mould. The conduits are finally immersed in ethanol to produce the permanent β-sheet conformation and freeze-dried prior to their use.

### Macro and microstructure of the conduits

**Fig. 1:**
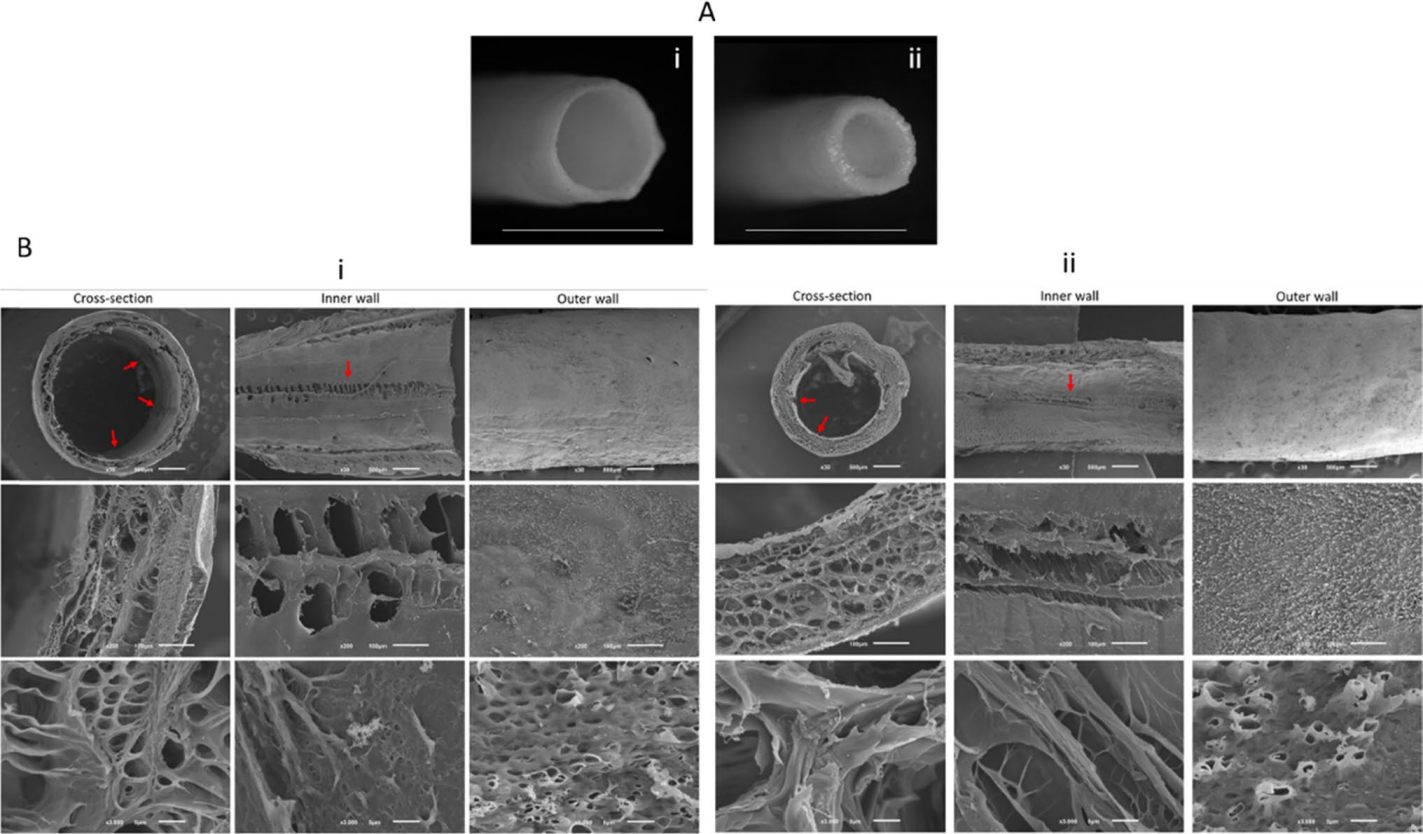
**A** Stereomicroscope representative images of thin (i) and thick (ii) conduits with inner pattern. Scale bar = 1 cm. **B** SEM microphotographs of the thin (i) and thick (ii) conduits with inner pattern cross-section (first column), inner wall (second column) and outer wall (third column), taken at a magnification of × 30 (first row, scale-bar = 500 μm), × 200 (second row, scale-bar = 100 μm) and × 3000 (third row, scale-bar = 5 μm)

Stereomicroscopic and SEM images are used to characterize the structure of the conduits. Stereomicroscopic image analysis shown in Fig. 1A and S1A reveals a difference on conduit wall thickness, and from SEM images of conduit cross-section (Fig. 1B and S1B, Column 1), wall thickness can be obtained; ~ 200 μm for thin conduits and ~ 400 μm for thick conduits, obtaining the same wall thickness for conduits with inner pattern. Total conduit diameter is ~ 2.5 mm, which does not vary between different conduits. In Fig. 1B and S1B, the inner (Column 2) and outer (Column 3) microstructures of the conduits are shown. The pattern is formed in the inner wall (red arrows) when the mould has longitudinal patterns. Moreover, the images of the inner and outer microstructure show that conduit walls have pores which are highly important for nutrient and waste exchange. In PNR, the porosity plays an important role for micro blood vessel penetration in order to supply nutrients and oxygen, as in long nerve gaps, these elements cannot diffuse through the longitudinal axis of the graft. If pore size is large, non-neural cells can enter into the lumen and form scar tissue, and its excess leads to an impaired nerve regeneration [40]. Pore size needs to be optimal to achieve a balance between the outward diffusion of growth factors and scar tissue formation. Cross-section SEM images reveal a highly interconnected porous microstructure for the four types of SF conduits.

### Conformation of the constructs

**Fig. 2:**
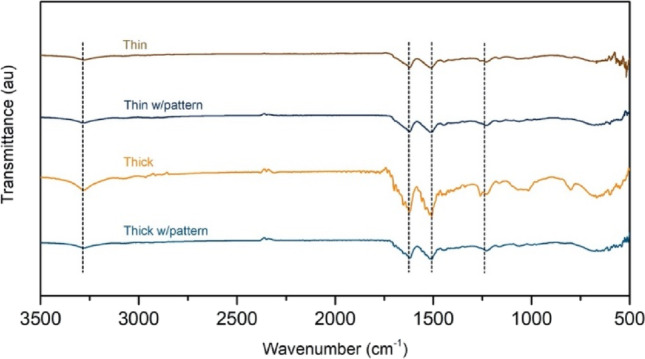
FTIR spectra of the different silk fibroin conduits. Similar spectra are obtained regardless of the thickness of the wall and the presence of patterns in the inner wall. The spectra show characteristic peaks at 1627 cm^−1^ (amide I), 1520 cm^−1^ (amide II), 1262 cm^−1^ (amide III) and 3300 cm^−1^ (N–H stretching vibration).

β-sheet conformation is related to semi-crystalline state and low water solubility. The conformation of the developed conduits is shown in Fig. 2 and was evaluated by ATR-FTIR to confirm the transition from amorphous to crystalline state. The intermediate structure in the form of hydrogel is characterized by an amorphous state; however, all final conduits displayed absorbance peaks at 1627 and 1520 cm^−1^, which are characteristic of β-sheet conformation (amide I and II band) [41]. Moreover, at 1262 cm^−1^, a shoulder appears, which corresponds to the amide III band [42]. The major silk fibroin band is centred at 3300 cm^−1^, which is the stretching vibration of the NH moiety of the amide group involved in both inter- and intramolecular hydrogen bonds [43].

### Mechanical properties of the conduits

Mechanical properties have been demonstrated to be a highly important regulator of axonal regeneration and elongation [44]. The surrounding musculoskeletal system exerts mechanical loads to the nerves, due to their mission to conduct action potentials [45]. In resting position, peripheral nerves are subjected to tensile loads, which makes them experience around 12% of mechanical strains while resting [46]. So, at this strain, conduits have to be able to elongate. Thin conduits need to be subjected to a tensile stress of 0.29 ± 0.03 MPa to achieve 12% of strain, and when conduits have inner longitudinal patterns, the value increases to 0.34 ± 0.02 MPa. In the case of thick conduits, a higher tensile stress needs to be applied. It has to be of 0.42 ± 0.04 MPa and 0.50 ± 0.03 MPa for thick conduits and thick conduits with inner pattern, respectively. These results indicate that the conduit with thicker walls and with longitudinal pattern has the higher resistance when this load is applied.

**Fig. 3:**
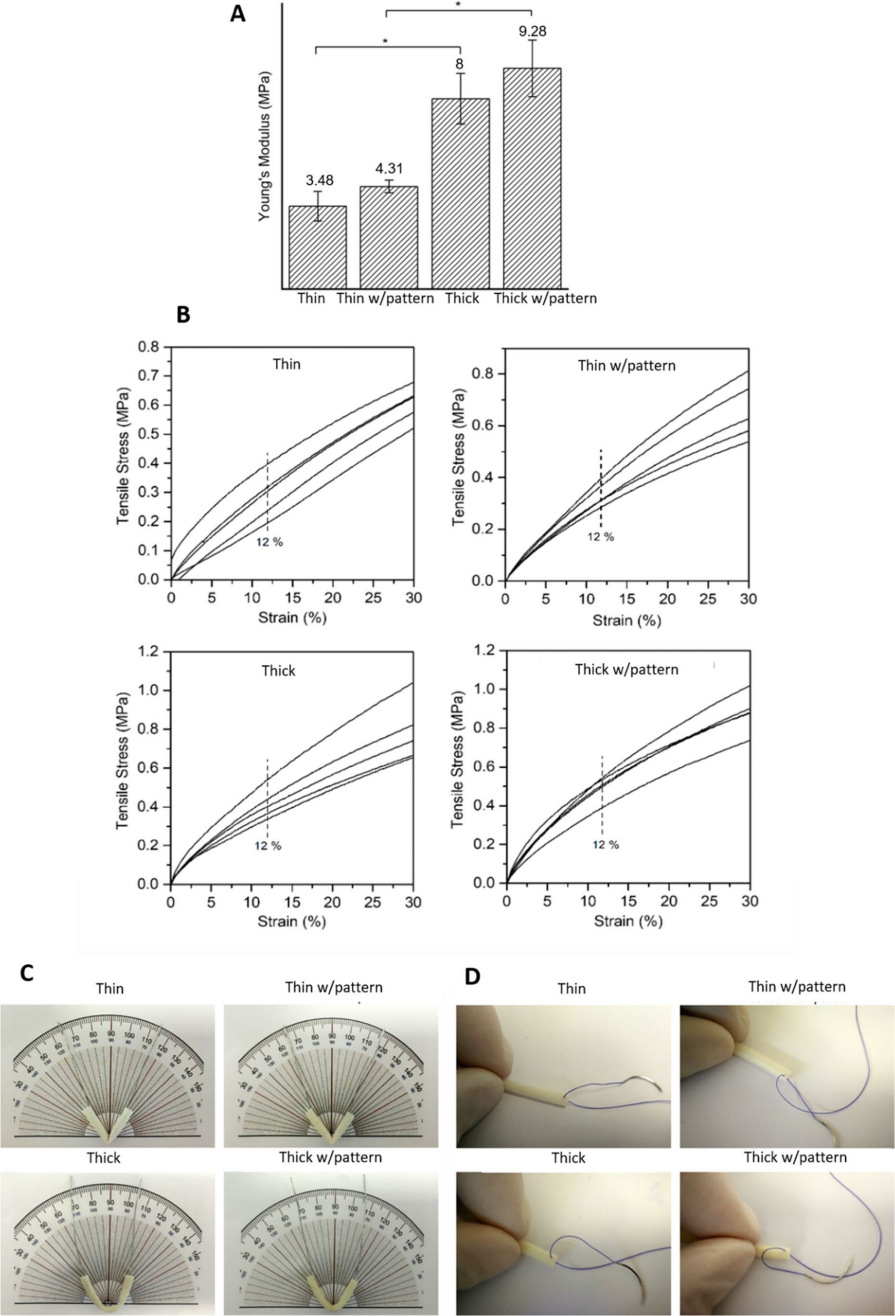
Mechanical properties of the conduits. **A** Tensile modulus of the thin and thick conduits with and without pattern. Quantitative data is presented as mean and error bars (*n* = 5), where *p < 0.05. **B** Strain (%) as a function of tensile stress (MPa) applied in the conduits, where the dotted line represents the point where 12% of strain is achieved. **C** Kinking tests of thin, thin conduit with pattern, thick and thick conduit with pattern. **D** Suturability test performed on the conduits inserting a 4-0 surgical suture

Nerve guidance conduits should not outperform the mechanical properties peripheral nerves have; it can cause discontinuous elasticity between the conduit and the growth of the new tissue [47, 48]. Literature has reported that porcine peroneal and tibial nerves have a Young’s modulus of around 7 MPa, a value which is in consonance with the values we obtained for our conduits, which are in the same order of magnitude [49]. Figure 3 summarizes the mechanical properties of the conduits. Strain-stress curves are represented for the 5 conduits tested for each condition in Fig. 3B, and with the dashed line, the tensile stress at 12% of strain is highlighted. From these curves, Young’s modulus is calculated though the tangent method, and the results are shown in Fig. 3A. Stiffest conduits are the thick ones with pattern, with a Young’s modulus of 9.28 ± 1.20 MPa, but the difference with the modulus of thick conduits without pattern is not significative, being for these conduits of 8 ± 1.06 MPa. Results between thin and thick conduits are significative even if conduits have or not longitudinal patterns. The stiffness of thin conduits is lower, 3.48 ± 0.61 MPa and 4.3 ± 0.27 MPa without and with pattern, respectively. Again, conduits with pattern exhibit a higher modulus, even if the difference is not significative.

Conduits might be also placed in flexible body parts, what might make them bend, and occasionally occlude, which is undesired for this application. Kinking resistance is evaluated blending the conduits to 50°, as the FDA-approved NeuraGen conduit collapses at this angle of blending [50]. Photos are taken to the experiments, shown in Fig. 3C. When conduit wall is thin, the collapse point is achieved before blending the conduit to 50°, but when the conduits have a thick wall, even without or with inner wall pattern, they do not blend at 50°, and the lumen does not get occluded. Moreover, the conduits need to be resistive to sutures if they are going to be used as implants, and they need to hold structural integrity and stability during surgery. To test if these conduits are suitable for grafting, suturability tests are performed, and it is demonstrated they are appropriate as can be seen in Fig. 3D.

### Bioactivity of the conduits

Nerve guidance conduits cannot calcify when they are implanted considering their application in peripheral nerve repair, and to test the developed conduits, their apatite-forming ability is determined by soaking them in simulated body fluid (SBF) solution [51]. Table 1 shows the weight percentage of Ca and P ions detected on the surface of conduits through energy-dispersive spectroscopy (EDS), whose value is 0 or non-significative, confirming that all the tested conduits do not have the apatite-forming capacity [52].

**Table 1:** EDS results of conduits tested after 30 days of interaction in SBF, revealing the elemental composition and their relative abundance present in the surface. wt% stands for percentage by weight and σ stands for standard deviation

	Thin wall		Thick wall		Thin wall with pattern		Thick wall with pattern	
	wt%	σ	wt%	σ	wt%	σ	wt%	σ
C	51.9	1.5	49.4	1.7	52.7	1.4	52.3	1.5
O	25.3	0.9	26.4	1.1	25.2	0.8	24.8	0.9
N	22.2	2.0	21.3	2.4	21.5	1.9	21.9	2.0
Si	0.1	0	0.5	0.1	0	0	0.1	0
Na	0.3	0.1	0.8	0.1	0.2	0	o.0	0.1
Cl	0.3	0	1.4	0.1	0.2	0.1	0.5	0
Ca	0	0	0.1	0	0	0	0.1	0
K	0	0	0.1	0	0	0	0	0
P	0	0	0	0	0	0	0	0

### In vitro biological activity of the conduits

**Fig. 4:**
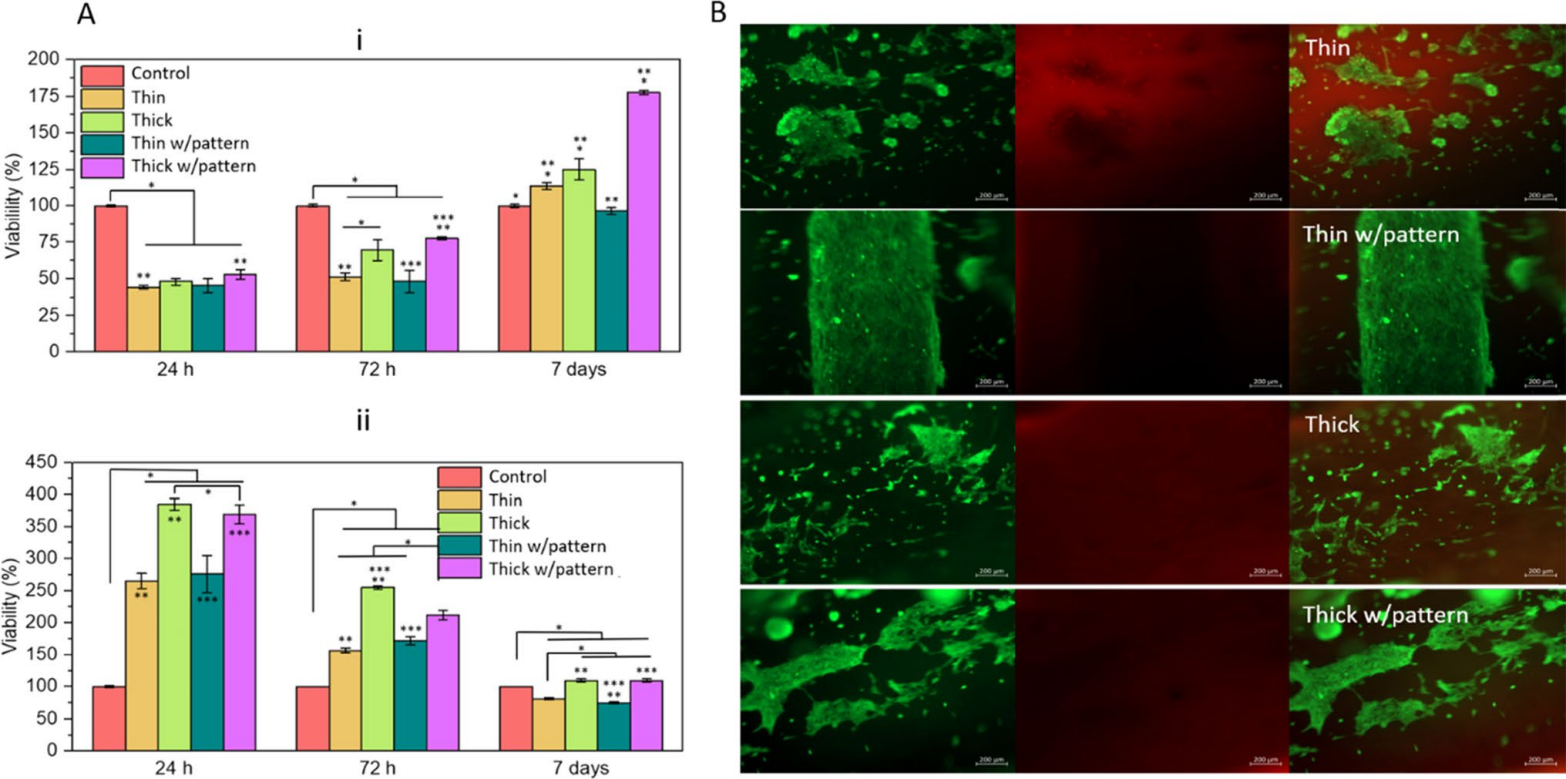
**A** Qualitative evaluation of iSCs (i), and BJ fibroblast (ii) viability when they are cultured on top of thin and thick wall SF NGCs without and with inner longitudinally aligned pattern. As control, cells are seeded on adherent culture plates. Data is normalized to the control group at each time point and presented as mean and error bars (*n* = 3), where *, **, *** *p* < 0.05. **B** Calcein-AM/Propidium iodide staining demonstrating the presence of viable/dead SCs adhered to the interior surface of the conduits after 24 h of culturing. First column are images of green channel, which represent the live cells, capable of migration. The second column is red channel images, representing dead cells. Third column shows the merged images of green and red channels

It is also crucial to investigate whether NGCs would provide a supportive luminal surface for proliferation of Schwann cells (SCs) after an injury, as these cells have key roles in the regeneration process [53]. For this purpose, SCs are directly seeded in the interior surface of the studied conduits and their viability is evaluated; results are plotted in Fig. 4A (i). Regarding the thick conduits, after 72 h of culture, the difference is significative if they are compared to thin conduits; these results are same when there is inner pattern or not. After 7 days, however, the proliferation is higher when cells are cultured on top of thick conduits with inner pattern. Even if results are improved for thick conduits, when NGC wall is thinner, the results are also positive if they are compared to the control. Same in vitro, the experiment is performed with BJ fibroblasts to study their proliferation on top of our SF NGCs. Figure 4 A (ii) shows the results at 24 and 72 h and 7 days of culture. Again, an improved proliferation is observed at day 7 when cells are cultured on top of thick conduits, if compared to thin wall NGCs. Also, after 7 days of culture, results of viability are significantly lower if compared to the control group. The presence of the inner pattern has been demonstrated to gift the NGCs with a higher biocompatibility for both studied cell types. These results can indicate that the patterning and guiding of cells with the pattern can enhance their growth and attachment to the conduit. Cells can also sense the stiffness of the surface, and it was seen that the Young’s modulus is also better when the conduits have patterns, which can also have a positive consequence on cell proliferation [54]. As previously discussed, the stiffest conduits are the thick ones with pattern, but the stiffness of thick wall conduits without pattern is comparable, as the difference is not significative. Same behaviour is observed within thin conduits. Compared to the thick ones, they are significantly less stiff, but between patterned and not patterned thin conduits, the difference is not significative. As discussed in the work by Gu et al., SCs can sense the stiffness of the substrate [55], and in our case, we see higher SCs proliferation after 7 days of culture on the stiffest conduits, which is the patterned thick conduit. In the case of BJ fibroblast proliferation, after 7 days of culture, we do not see any difference on cell viability; however, in both cases, the rate is the highest.

Figures 4 B and S2 show fluorescence microscopy images of iSCs cultured on top of SF conduits for 24 and 72 h and 7 days, respectively. Very few dead cells, labelled with propidium iodide in red colour, are detected, proving once again the good biocompatibility of the SF conduits. After 24 h of iSCs culturing, it can be observed that cells grow forming longitudinally aligned pattern, following the grooves that have been produced on the inner surface of the conduits.

**Fig. 5:**
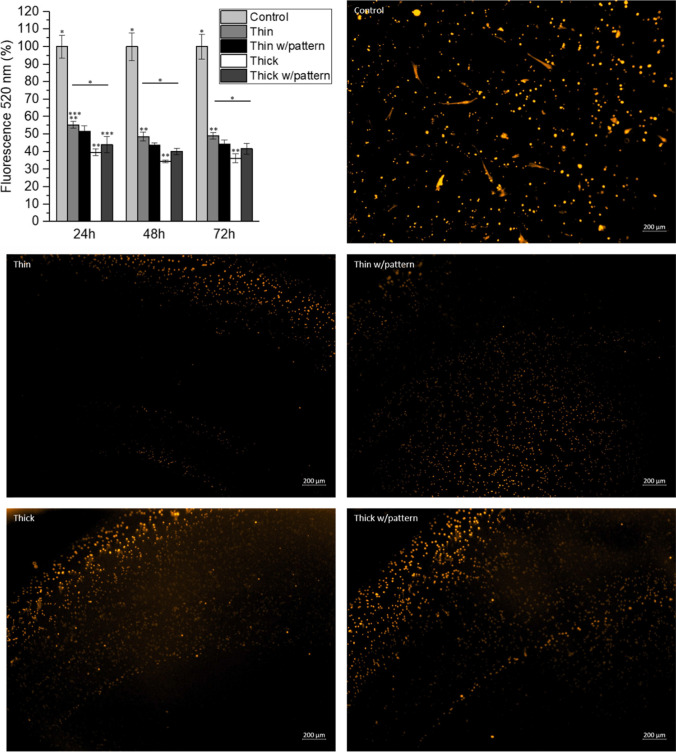
Infiltration study of BJ fibroblasts: fluorescence quantitative measurement of infiltrating cells through the conduits shown in the bar-plot, and data is normalized to the control group at each time point and presented as mean and error bars (*n* = 3), where *, **, ***p < 0.05. Fluorescence images are taken at 48 h of Red tracker labelled BJ fibroblasts that have migrated through the Boyden chamber

It is highly important to avoid cells that can promote scar formation entering to the lumen of the conduit. If fibroblasts enter and form scar tissue, nerve regeneration would be impaired [40]. As can be seen in the graph of Fig. 5, BJ fibroblasts cannot infiltrate through none of the conduits; the control is referred to BJ fibroblasts seeded directly on top of the membrane of the Boyden Chamber. These results are confirmed qualitatively by fluorescence imaging; images of the bottom of the well are taken to observe BJ fibroblasts that passed through the membrane. Figure S3 shows fluorescent images of the infiltration studies at higher magnification. Images referred to SCs grown on top of conduits are only showing the reflectance of the pores of the membrane of the Boyden Chamber. These results indicate that fibroblasts would not be able to enter to the lumen of the developed conduits; there would not be scar formation inside the graft, without inferring on the nerve regeneration process.

## Experimental

### Materials

SF purification. The purified SF is prepared as previously described by Yan et al. [56] Briefly, SF is first separated from sericin, the other main protein in the cocoons. The cocoons are immersed in a 0.02 M boiling sodium carbonate solution for 1 h, followed by rinsing with abundant distilled water. The obtained SF is then immersed in a 9.3 M lithium bromide solution at 70 °C until its complete dissolution and dialyzed (benzoylated dialysis tubing, MWCO: 2 KDa) for 48 h to remove impurities. The purified SF is concentrated in 20 wt% poly (ethylene glycol) solution for at least 6 h. The final concentration is determined by weighting the dry product, and the SF solution is stored at 4 °C for a maximum of 5 days.

Fabrication of the conduits. SF solution is diluted to 16 wt% with distilled water, after which it is mixed with horseradish peroxidase solution (HRP type VI, 0.84 mg·mL^−1^, 100 μL) and hydrogen peroxide solution (H_2_O_2_, 0.36 wt%, 65 μL; Panreac, Barcelona, Spain) [56]. The previous mixture is injected within the space between two concentric cylinder moulds, where the outer cylinder has a constant diameter of 4 mm and is made of polypropylene. Inner cylinders made of iron oxide are purchased from the Neves & Neves Metalomecânica, Lda (São Mamede do Coronado, Portugal). Inner cylinders have a diameter of 2 and 3 mm, which leads to conduits with thick and thin walls, respectively. Moreover, cylinders with longitudinally aligned patterns are used, in both, thick and thin formats. System is incubated at 37 °C in order to induce gelation for a period of 30 min. After gelation, a quick immersion in liquid nitrogen is needed to remove the outer mould, followed by an immersion in ethanol to induce permanent β-sheet conformation and to remove the inner mould. Conduits are then freeze-dried after freezing at −80°C.

### Methods

Stereomicroscopic evaluation. For macroscopic evaluation, conduits are viewed under a Stereo Microscope with Lamp (Schott KL 200, model Stemi 1000, Zeiss, Germany).

Scanning electron microscopy (SEM). The morphology and microstructure of the conduits are evaluated by SEM (model S360, Leica, Cambridge, England). Conduits are previously coated with gold for imaging.

Fourier-transform infrared spectroscopy (FTIR). The chemical composition and conformation of the conduits is addressed in an IRPrestige-21 (Shimadzu, Japan) by attenuated total reflectance (ATR) FTIR spectroscopy. The spectra are obtained from an average of 32 scans between 3500 and 500 cm^−1^ taken at 4 cm^−1^ resolution.

Mechanical testing. An Instron universal mechanical testing machine (Model 5540, USA) is used to study the mechanical properties of the conduits following the ASTMC749-08 standard method. A load cell of 50 N is used. Samples are fixed using a grip and are stresses with a cross-head speed of 2 mm min^−1^ until rupture is reached. Strain-stress curves are obtained for 5 hydrated samples in PBS 10 mM at 37 °C overnight, with 3-mm length to determine the tensile Young’s modulus by the tangent method.

Kinking tests are performed with 3-cm length conduits by bending them up to 50° in a metallic flexible 0.5-mm wire and assessing macroscopically the bending point.

The suturability is assessed by means of inserting a 4-0 surgical suture at the place where peripheral nerve suturing would be performed, which is 2 mm from the end of the conduit, considering its long axis and strain to the rupture point.

In vitro bioactivity test. For the bioactivity study, an acellular simulated body fluid (SBF) solution is prepared as described by Kokubo et al. [51] The SBF contains the same ions as human blood plasma, at nearly equal concentrations. Conduits are immersed in freshly prepared SBF for 1, 15 and 30 days at 37 °C, and then they are rinsed with distilled water and left to dry. The presence or absence of a calcium-phosphate layer on their surface is determined using SEM equipped with an energy-dispersive spectroscope (EDS) (INCAx-Act, PentaFET Precision, Oxford Instruments), at an accelerated voltage of 15 kV.

Cell culture of immortalized human Schwann cells (iSCs) and immortalized human skin fibroblasts (BJ). Immortalized Schwann cells (iSCs, sNF96.2, ATCC) are cultured in high glucose Dulbecco’s modified Eagle’s medium supplemented with 10 % FBS, 1% penicillin/streptomycin and 1% sodium pyruvate, in non-coated cell culture flasks. BJ fibroblasts (BJ, CRL-2522, ATCC) are cultured in Eagle’s minimum essential medium, supplemented with 10% FBS and 1% penicillin/streptomycin and 1% sodium pyruvate. Cell’s medium is changed every 2–3 days and kept at 37 °C and 5% CO_2_.

Biocompatibility of the conduits. The viability of iSC and BJ fibroblasts is followed with Alamar Blue (AB), a dye that yields a fluorescent signal when incubated with metabolically active cells. The greater the AB dye reduction, the higher is the cells viability. Sterilized conduits are cut at 14 mm and in halves for direct cell seeding on the inner concave surface. Conduits are placed in non-adherent culture plates, while adherent culture plates are used as both cell type growth control. A 20-μL drop at 105 cells mL^−1^ is placed on the conduits, and cells are left to adhere for 1 h. Then, 500 μL of medium is added to each well. As controls, 500 μL of cells at a concentration of 104 cells⋅mL^−1^ is seeded. After 1, 3 and 7 days in culture, specific cell culture medium containing 20% AB was added to the different culture wells. The system is incubated for 3.5 h, and fluorescence is monitored at 590-nm emission wavelength (excitation wavelength 530 nm), using a FL 600, Bio-Tek Instruments microplate reader. The PBS is used to remove the excess of AB reagent, and fresh culture medium is added in its place after each AB determination. Corrections to control group of cell density and available area for them to grow are made.

The qualitative evaluation of viability of iSC within the conduits is followed with a live/dead assay at 24 and 72 h and 7 days. Briefly, cell-laden conduits are washed three times with PBS and incubated for 30 min, at 37 °C in dark with 10-6 M Calcein AM and 1.5 × 10^−6^ M propidium iodide. Samples are then washed with PBS three times and imaged using a transmitted and reflected light microscope (Axio Imager Z1m, Zeiss, Jena, Germany).

In vitro cellular permeability assay (using a modified Boyden chamber). A cell migration assay is designed to verify the cellular permeability of the SF conduits to BJ fibroblasts, using HTS Corning FluoroBlok Cell Culture Inserts (24-well) with an 8-μm pore size (Becton Dickinson, USA). For direct cellular seeding, conduits with 4 mm in length are cut longitudinally (to fit the insert) and used in halves. Conduits are first hydrated for 1 h in sterile PBS. BJ fibroblasts are prelabelled with Cell Tracker Red (Invitrogen, CA, USA) for imaging, and Cell Tracker Green (Invitrogen, CA, USA) for plate reader measurements for 30 min (5 × 10^−3^ M), and then seeded directly in the internal and concave surface of the conduit at a cell density of 8 × 10^3^ cells per conduit. As control, cells are directly seeded in the upper well. Cellular adhesion is allowed to occur in a normal non-adherent 24-well plate for 3 h, using a serum-free medium. Cell-laden constructs are then moved to the upper chamber of the HTS Corning FluoroBlok Cell Culture Inserts, making sure the external and convex surface of the conduit is in contact with the insert surface. In the upper chamber, where the construct is placed, MEM serum-free medium is added and in the lower chamber, MEM medium supplemented with 40 vol% FBS is added, in order to serve as a chemoattractant gradient. As a positive control, the same cellular density is directly seeded in the upper chamber. At different time points (24, 48 and 72 h), fluorescence intensity from the bottom (basal side) is measured using a microplate spectrofluorometer (FL 600, Bio-Tek Instruments) in area-scan bottom-reading mode, at excitation/emission wavelengths of 485/520 nm. Fluorescence images of cells that migrated through the conduit wall after the last time-point (48 h) are collected using an inverted fluorescence microscope (model TCS SP8, Leica, Germany).

Statistical analysis. The statistical ANOVA analysis is performed in OriginPro 2016 software. Fisher’s tests are performed to determine statistically significant differences with a *p* < 0.05.

## Conclusions

Nerve guidance conduits are of special interest as they can be used as substitutes to current ineffective approaches for PNR treatment after an injury occurs. The present work shows the synthesis of tubular conduits with inner longitudinally aligned patterns. The SF-based conduits have been demonstrated to be biocompatible and fulfil the mechanical properties required to be used in flexible body parts and stand tensile stress derived by it. Tuning the inner wall of the conduits, by using a different inner mould to achieve that, has demonstrated that the mechanical and biological outcomes can be enhanced. The developed nerve guidance conduits with inner longitudinally aligned pattern have shown to have improved mechanical properties, with a better Young’s modulus, and moreover, when the wall is thicker, their kinking resistance is also improved. Regarding the in vitro outcomes, enhanced cell proliferation, in both iSCs and BJ fibroblasts, was observed with the inner pattern. Also, in every case BJ fibroblasts cannot infiltrate through the SF conduits, avoiding scar tissue formation in the lumen and increasing the chances of success of the graft.

## Supplementary information

ESM 1

## Data Availability

The data presented in this study are available on request from the corresponding author. The data are not publicly available due to privacy restrictions.
