# Supplementary material for: Longitudinally aligned inner-patterned silk fibroin conduits for peripheral nerve regeneration
Source: In Vitro Model. 2023 Apr 18;2(5):195–205. doi: 10.1007/s44164-023-00050-3 (PMC11756464; doi:10.1007/s44164-023-00050-3)
Supplement: Supplementary file 1 — ESM 1 [file 44164_2023_50_MOESM1_ESM.docx]

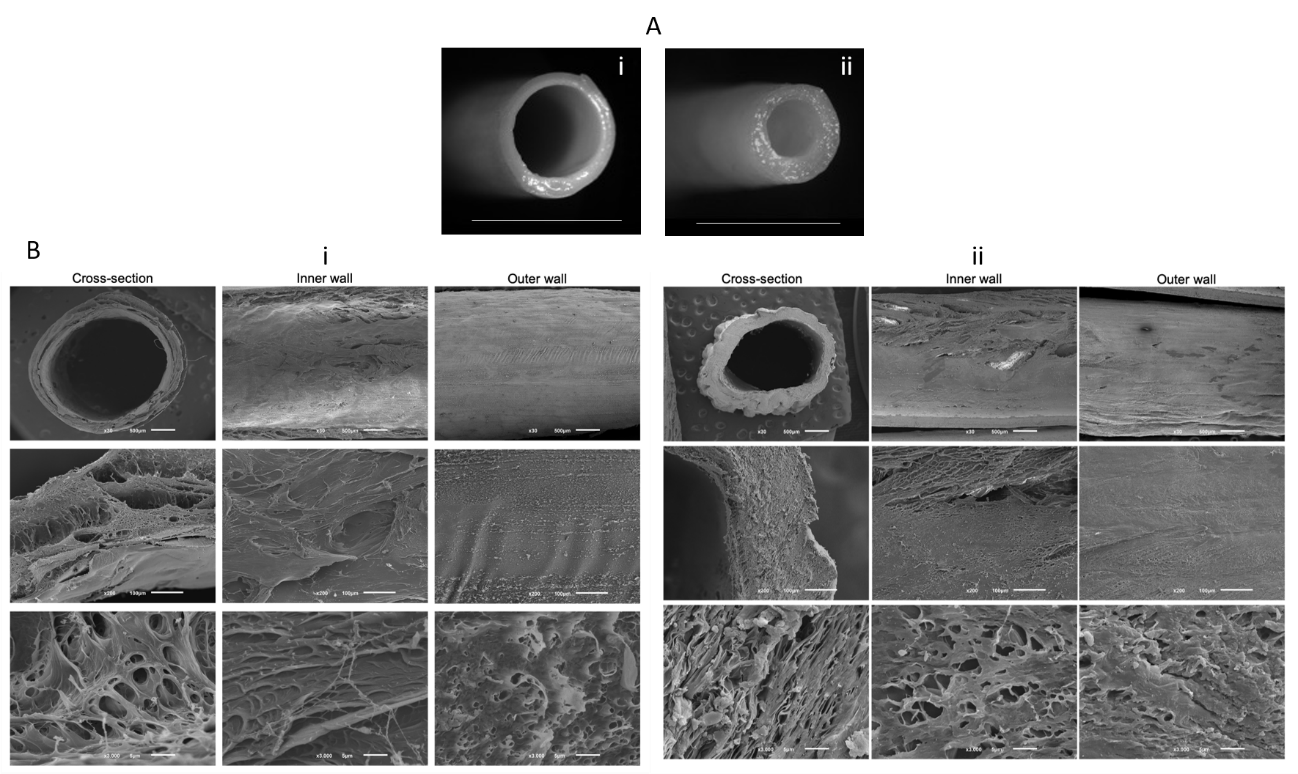


*Figure S1. A) Stereomicroscope representative images of thin (i) and thick (ii) conduits. Scale- bar = 1 cm. B) SEM microphotographs of the thin (i) and thick (ii) conduits cross-section (first column), inner wall (second column) and outer wall (third column), taken at a magnification of x 30 (first row, scale-bar = 500 µm), x 200 (second row, scale-bar = 100 µm) and x 3,000 (third row, scale-bar = 5 µm).*


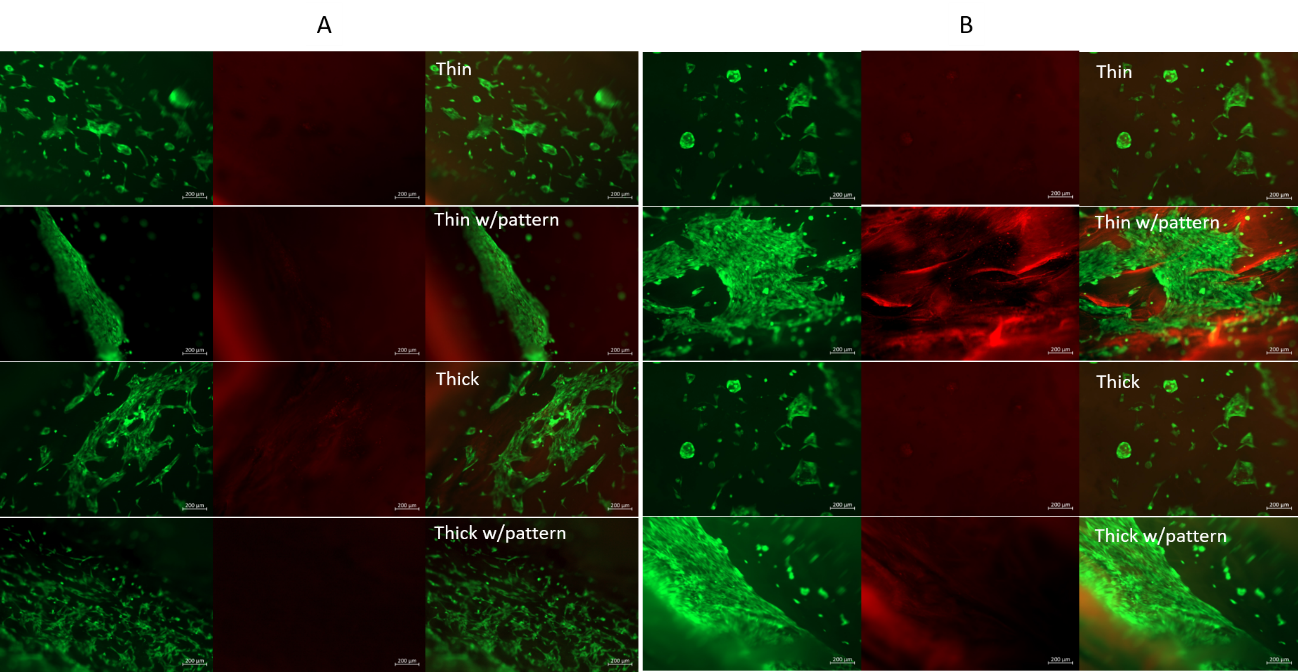


*Figure S2. Calcein-AM/Propidium iodide staining demonstrating the presence of viable/dead SCs adhered to the interior surface of the conduits. First column are images of green channel, which represent the live cells, capable of migrate. The second column are red channel images, representing dead cells. Third column shows the merged images of green and red channels. Images are taken at A) 72 h and B) 7 days.*


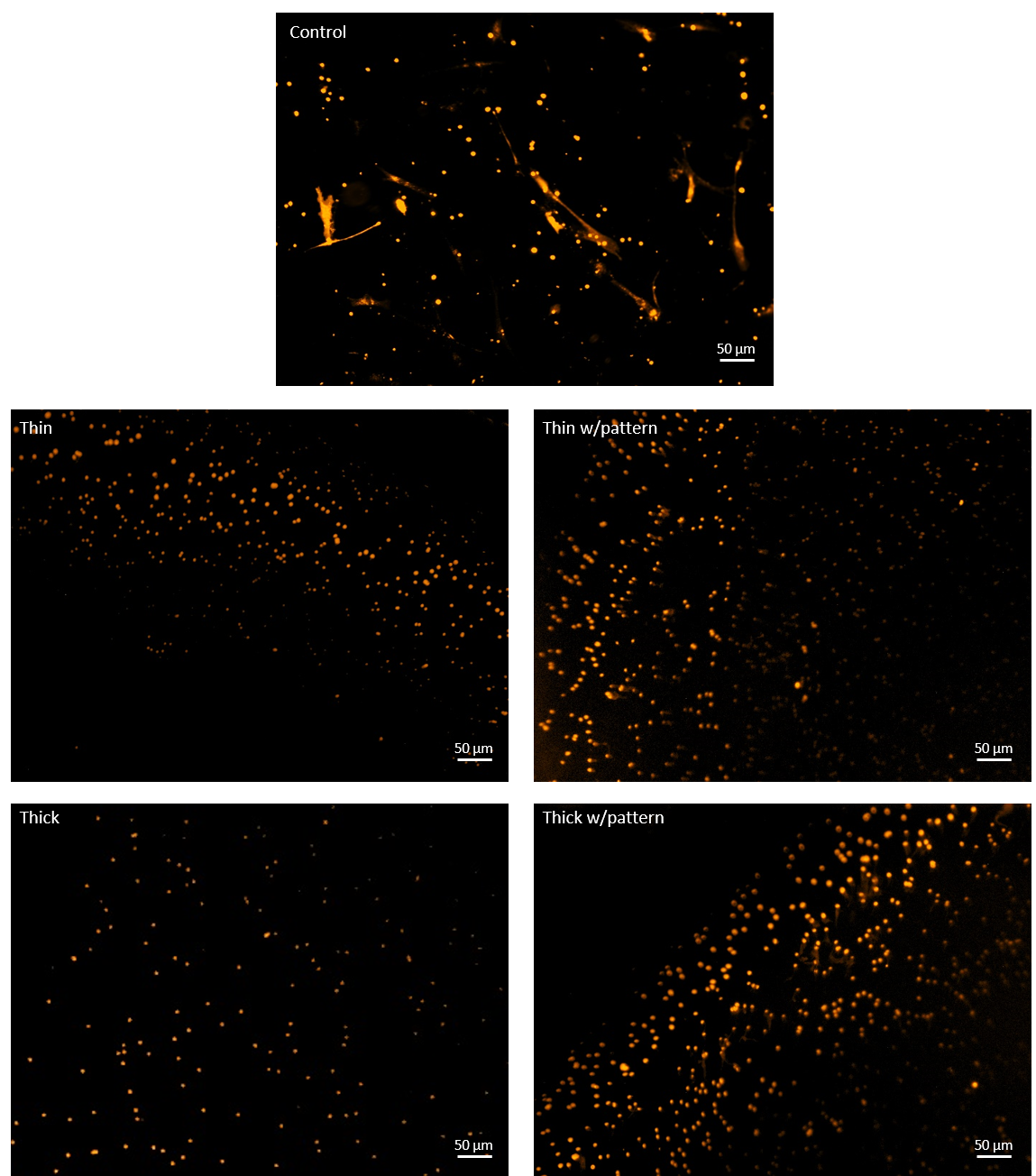


*Figure S3. Infiltration study of BJ fibroblasts: Images taken at 48 h of Red tracker labelled BJ fibroblasts that have migrated through the Boyden chamber.*
